# Melatonin-induced ApoE expression in mouse astrocytes protects endothelial cells from OGD-R induced injuries

**DOI:** 10.1038/s41398-020-00864-9

**Published:** 2020-06-08

**Authors:** Jun Xiang, Wen Zhu, Feng Yang, Zhong-Hai Yu, Min Cai, Xiang-Ting Li, Jing-Si Zhang, Wen Zhang, Ding-Fang Cai

**Affiliations:** 1 Department of Integrative Medicine, Zhongshan Hospital, Fudan University, 200032 Shanghai, China; 2 Laboratory of Neurology, Institute of Integrative Medicine, Fudan University, 200032 Shanghai, China; 3 Department of Traditional Chinese Medicine, the Sixth People’s Hospital Affiliated to Shanghai Jiao Tong University, 200233 Shanghai, China; 4 Department of Neurology, Shuguang Hospital Affiliated to Shanghai University of Traditional Chinese Medicine, 201203 Shanghai, China

**Keywords:** Molecular neuroscience, Pathogenesis, Molecular neuroscience, Pathogenesis, Molecular neuroscience

## Abstract

Stroke is a leading reason of death and long-term disability, and most studies mainly focus on efforts to protect neurons. However, failed clinical trials suggest that therapies against single target in neurons may not be sufficient and the involvement of endothelial cells and glial cells have been underestimated. Astrocytes are the major source of ApoE in the brain and endothelial cells express high level of ApoE receptors. Thus, ApoE may mediate the interaction between astrocytes and endothelial cells. To address whether and how ApoE-mediated astrocytes–endothelial cells interaction contributes to the pathogenesis of stroke, we used oxygen and glucose deprivation-reoxygenation (OGD-R) as a stroke model and investigated the effects of OGD-R on astrocytes-endothelial cell co-cultures in the current study. We find that OGD-R leads to various damages to endothelial cells, including compromised cell viability, increased ROS level, enhanced caspase activity, and higher apoptotic rate. Meanwhile, mouse astrocytes could secrete ApoE to activate PI3K/eNOS signaling in endothelial cells to prevent OGD-R induced injuries. In addition, OGD-R induces down-regulation of ApoE in astrocyte–endothelial cell co-cultures while melatonin restores astrocytic ApoE expression via pCREB pathway and protects endothelial cell in OGD-R treated co-cultures. Our study provides evidence that astrocytes could protect endothelial cells via ApoE in OGD-R condition and Melatonin could induce ApoE expression to protect endothelial cells.

## Introduction

Stroke is a leading reason of death and long-term disability in the world^1^. Ischemic stroke is caused by stenosis or occlusion of the blood vessels that supply oxygen and nutrients to brain tissues. As the brain tissues consume energy at high rate^2^, the insufficient blood supply leads to the disturbance of brain homeostasis, resulting in oxidative stress, excitotoxicity, inflammation, disruption of the blood-cerebrospinal fluid barrier, and infiltration of peripheral immune cells to the central region of ischemia^3^.

Although many cell types such as neurons, vascular endothelial cells, and glial cells are involved in the pathogenesis of stroke^4^, many studies mainly focus on efforts to protect and/or rescue neurons. Unfortunately, clinical trials to prevent ischemic neuronal death by blocking the detrimental pathways have failed^5^. It suggests that therapies against single target in neurons may not be sufficient and the involvement of endothelial cells and glial cells has been underestimated. Astrocytes play essential roles in the brain through the ion buffering, the uptake and synthesis of neurotransmitters, controlling cerebral blood flow, release of antioxidant substances, immunomodulation, and neurogenesis^6^. In addition, multiple investigations have revealed that astrocytes also contribute to the central ischemic brain stroke^7^. However, the interaction between astrocytes and endothelial cells in stroke are poorly understood.

ApoE is a member of the apolipoprotein family which is essential for the cholesterol metabolism^8^. Human ApoE gene has three major alleles: ApoE2, ApoE3, and ApoE4. The wild-type ApoE3 is essential for synaptic plasticity, cell signaling, lipid transport and metabolism, and Aβ clearance^9^. Not coincidently, astrocytes are the major source of ApoE in the brain^10^ and endothelial cells express high level of ApoE receptors^11^. Thus, ApoE may mediate the interaction between astrocytes and endothelial cells. To address whether and how ApoE-mediated astrocytes–endothelial cell interaction contributes to the pathogenesis of stroke, we used oxygen and glucose deprivation-reoxygenation (OGD-R) as a stroke model and investigated the effects of OGD-R on astrocytes–endothelial cell co-cultures in the current study. We find that OGD-R leads to various damages to endothelial cells while mouse astrocytes could secrete ApoE to protect endothelial cells. In addition, Melatonin could induce ApoE expression in mouse astrocytes and shows protective effects in OGD-R treated co-cultures. Thus, our study reveals a novel interaction between astrocytes and endothelial cells under OGD-R condition and its underlying mechanism.

## Materials and methods

### Cell culture

Our study was approved by the Institutional Ethics Committee of Zhongshan Hospital, Fudan University. The primary astrocyte cultures were obtained from P1 pups of C57BL/6 mouse as described previously. Briefly, mice were decapitated and brain was dissected to obtain the cortex. Cortex tissues were minced with cold phosphate-buffered saline (PBS, pH 7.2–7.4), digested with 0.25% trypsin at 37 °C for 6 min, resuspended as a single-cell suspension in culture medium including DMEM/F-12 medium (Gibco, Gaithersburg, MD, USA) and 10% fetal bovine serum (FBS; Gibco, Gaithersburg, MD, USA) and seeded into a 25-cm^2^ culture flask pre-coated with Poly-d-lysine. Cells were maintained at 37 °C in a 95% humid atmosphere with 5% CO2. Microglia and other non-adherent cells were removed by shaking the culture flasks at 200 rpm for 2 h. Astrocyte cultures from day in-vitro (DIV) 7 to DIV10 were detached with 0.25% trypsin + 0.02% EDTA in PBS and plated at a density of 105 cells per 13 mm diameter coverslip in 24-well plates. Cells were cultured for 24 h to allow adhesion to coverslips. The purity of the astrocyte cultures was determined by staining cultures with GFAP antibody and DAPI. The purity was calculated using the number of GFAP positive cells divided by the number of DAPI nucleus. The purity is above 95%. Human umbilical vein/vascular endothelium cell line (HUVEC) was from ATCC (CRL-1730 and maintained in F-12K Medium with 10% fetal bovine serum (FBS).

### Co-cultures

The co-culture system consists of lower and upper chambers which were separated by a selectively permeable membrane with 0.4-μm-diameter pores (Corning, Transwell 3450). The primary mouse astrocytes were plated in the lower chamber and maintained to 10 days in vitro (DIV10). HUVEC cells were plated in the upper chamber and were co-cultured with astrocytes to simulate the Central Nervous System (CNS) microenvironment.

### Oxygen and glucose deprivation-reoxygenation

Oxygen and Glucose Deprivation-Reoxygenation (OGD-R) model was established as follows. Astrocytes were washed twice with OGD medium (DMEM without glucose) and maintained in OGD medium. Then, the cells were placed in a modular incubator chamber which was flushed with a 95% N2/5% CO2 gas mixture at 3 L/min for 30 min at room temperature. The chamber was then sealed and placed in a 37 °C container. After 2.5 hours of OGD, the cells were incubated in DMEM with glucose (without fetal bovine serum) for an additional 6 h of reoxygenation under normal conditions before subsequent analysis.

### ApoE over-expression lenti-virus

The coding sequences of mouse ApoE were cloned into pLenti-EGFP plasmid and packaged and amplified in HEK293T cells. HEK293T cell line was maintained in Dulbecco’s Modified Essential Medium (DMEM) with 10% FBS. Cells were cultured in a humidified atmosphere with 5% CO2 at 37 °C. Astrocytes were infected at an MOI of 10.

### Western blot

Proteins were extracted from cultured cells or culture medium using sodium dodecyl sulfate lysis buffer (2% sodium dodecyl sulfate, 10% glycerol, 0.1 mM dithiothreitol, and 0.2 M Tris–HCl, pH 6.8). Protein samples were resolved by SDS–PAGE and detected with indicated antibodies. The protein bands were quantified by densitometry analysis using Image J software and the intensity of each target protein was normalized by Tubulin intensity. Following antibodies were used: ApoE antibody from abcam (ab1907), LRP1 antibody from abcam (ab92544), LRP8 antibody from abcam (ab108208), LDLR antibody from abcam (ab52818), MT1 antibody from alomone (AMR-031), MT2 antibody from alomone (AMR-032), Akt antibody from CST (4691), Phospho-Akt (Ser473) antibody from CST (9271), eNOS antibody from abcam (ab76198), and Phosphor-eNOS (S1177) antibody from abcam (ab215717).

### Quantitative real-time PCR

Total RNA (1 μg) was reverse-transcribed into cDNA with random-hexamer primer mix using M-MLV Reverse Transcriptase (Promega, Madison, WI, USA) according to the manufacturer’s instructions. qRT-PCR was performed on a Rotor-Gene Q instrument (Qiagen) with cDNA, gene-specific primers and SYBR Premix Ex Taq (TaKaRa, Shiga, Japan). Following primers were used: ApoE forward: 5′-CTGACAGGATGCCTAGCCG-3′, ApoE reverse: 5′-CGCAGGTAATCCCAGAAGC-3′. Actin forward: 5′-GGCTGTATTCCCCTCCATCG-3′, Actin reverse: 5′-CCAGTTGGTAACAATGCCATGT-3′.

### Measurement of reactive oxygen species

After OGD-R and indicated treatment, astrocytes were washed with PBS and loaded with DCFH-DA (5 μM) from Reactive Oxygen Species Assay Kit (Beyotime Institute of Biotechnology, Jiangsu, China) for 40 min. After a wash with PBS, cell suspensions were collected into a 96-well flat bottom black plate to determine the relative fluorescent intensity (RFI, λex 485 nm, λem 535 nm) by Tecan GENios fluorescence microplate reader. The DMEM medium was used as blank. The RFI over control was calculated as the measured reactive oxygen species (ROS) production levels.

### CCK-8 assay

The cell viability was assessed by CCK-8 assay. In brief, 100 μl of media containing 5 × 103 cells was added to each well of a 96-well microplate. In all, 10 μl of Cell Counting Kit-8 reagent was added to each well and place the palte in a CO2 incubator for 1–4 h. Measure an absorbance on a microplate reader using a 450-nm filter. Cell viability was presented as the percentage of cell viability compared to the control group. Each experiment was repeated in triplicate using three independent cultures.

### Preparation of conditioned medium from astrocytes

The medium were replaced by normal medium without serum and astrocytes were further cultured for 48 h before the collection of conditioned medium. The conditioned medium (CM) were collected by centrifuge for 5 min at 1000×*g*. CM was concentrated using Amicon® Ultra 15 mL Centrifugal Filters (10kD). The concentration of ApoE in the conditioned medium was determined by ELISA kit and the final concentration of ApoE used to incubate cells is ~5–10 μg/ml.

### Annexin V-PE apoptosis assay

Cells were trypsinized, washed, and stained with Annexin V-PE Apoptosis kit (abcam, ab14155) in the dark for 15 min at room temperature. Then, the stained cells were analyzed by MoFlo XDP (Beckman Coulter, Inc).

### Caspase-Glo 3/7 assay

The Caspase-Glo® 3/7 Assay is a luminescent assay that measures caspase-3 and −7 activities in cell cultures. The assay was performed according to the manufacturer’s instruction. In brief, 100 µl of Caspase-Glo® 3/7 Reagent was added to each well of a white-walled 96-well plate containing 100 µl of blank, negative control cells or treated cells in culture medium. Cover the plate with a plate sealer and gently mix contents of wells using a plate shaker at 300–500 rpm for 30 s. Incubate at room temperature for 1 h and then measure the luminescence in a plate-reading luminometer.

### Statistical analysis

Statistical analysis was performed using GraphPad Prism software. All data were presented as mean ± SD and statistical analysis was performed by two-tailed Student *t* test for two groups and one way ANOVA with Newman–Keuls post hoc test for more than two groups. Statistically significant differences were defined as *P* < 0.05. For all, *P < 0.05, **P < 0.01, ***P < 0.001.

## Results

### Astrocyte-derived CM protects endothelial cell against OGD-R induced injuries

**Fig. 1:**
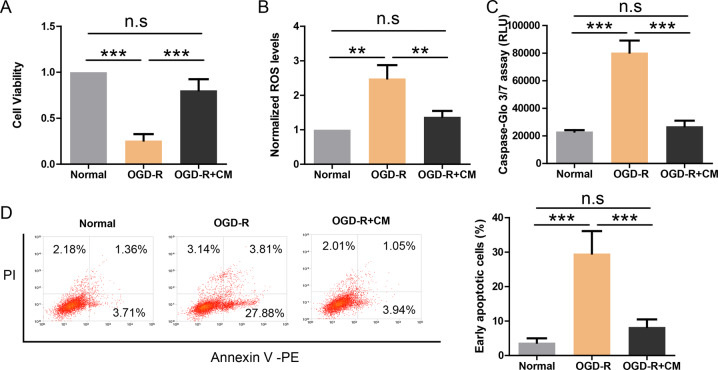
Astrocyte-derived CM protects endothelial cell against OGD-R induced injuries. **a** CCK-8 assay showing the cell viability of endothelial cells treated with OGD-R and astrocyte-derived CM (*n* = 4). **b** ROS production in endothelial cells treated with OGD-R and astrocyte-derived CM (*n* = 4). **c** Caspase-Glo 3/7 assay showing caspase activity in endothelial cells treated with OGD-R and astrocyte-derived CM (*n* = 4). **d** Flow-cytometry profiles of Annexin V/PI assay showing apoptosis of endothelial cells treated with OGD-R and astrocyte-derived CM (*n* = 4). **P < 0.01, ***P < 0.001.

We used OGD-R to recapitulate the effect of ischemia on in-vitro cultured endothelial cell line HUVEC and then evaluated potential effects with CCK-8 assay (Fig. 1a), ROS assay (Fig. 1b), Caspase-Glo 3/7 assay (Fig. 1c), and Annexin V-FITC apoptosis assay (Fig. 1d). The results showed that OGD-R treatment resulted in compromised cell viability, increased ROS level, enhanced caspase activity, and higher apoptotic rate. However, co-treatment of HUVEC cell with conditioned medium (CM) from normal mouse astrocytes could fully prevent these damages. It suggests that astrocytes might release protective cytokines.

### ApoE in CM mediates its protective effects on OGD-R treated endothelial cell

**Fig. 2:**
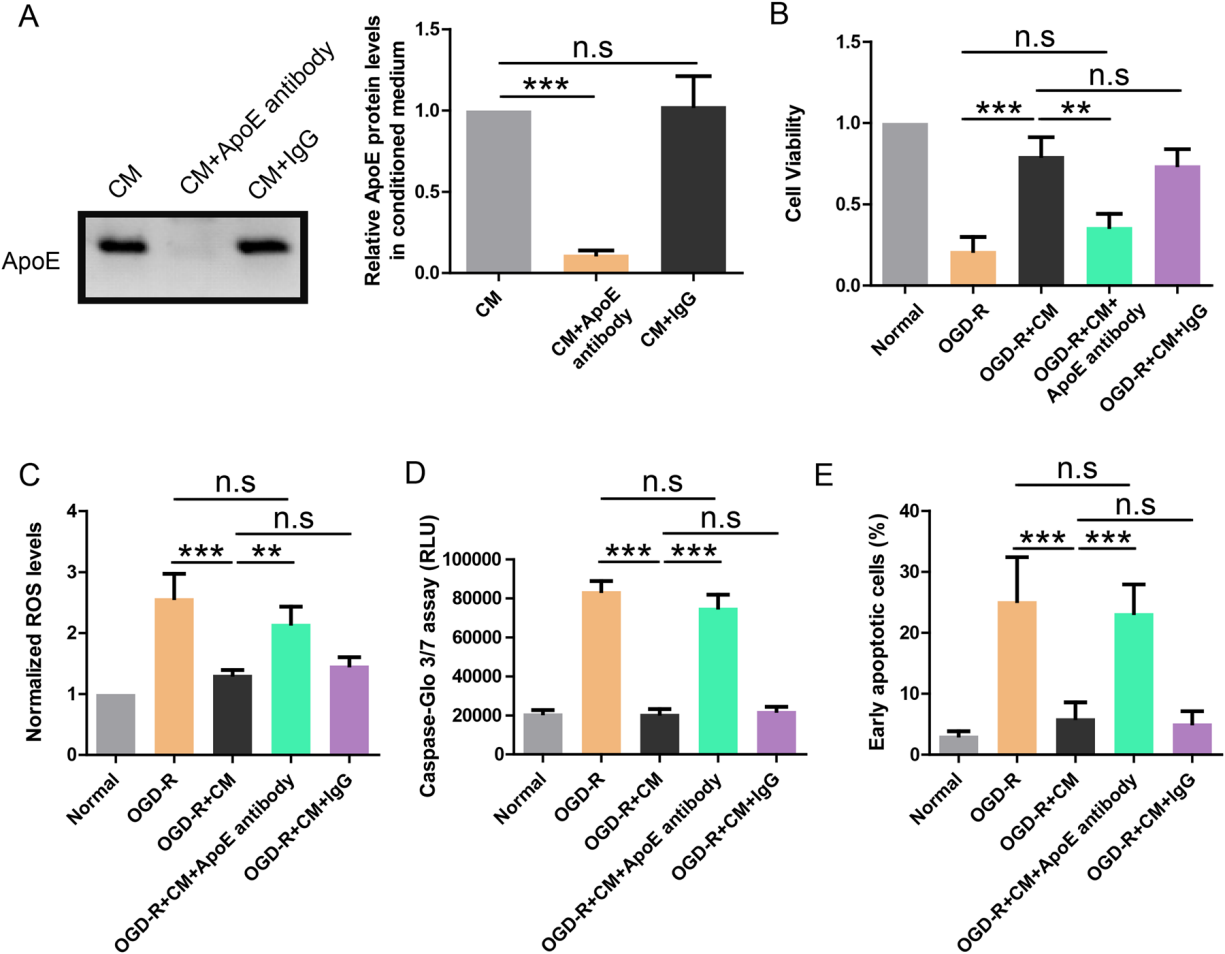
ApoE in CM mediates its protective effects on OGD-R treated endothelial cell. **a** Western blot showing the depletion of ApoE from astrocyte-derived CM by ApoE antibody. **b** CCK-8 assay showing the cell viability of endothelial cells treated with OGD-R and indicated CM (*n* = 4). **c** ROS production in endothelial cells treated with OGD-R and indicated CM (*n* = 4). **d** Caspase-Glo 3/7 assay showing caspase activity in endothelial cells treated with OGD-R and indicated CM (*n* = 4). **e** Apoptosis rate revealed by Annexin V/PI assay in endothelial cells treated with OGD-R and indicated CM (*n* = 4). **P < 0.01, ***P < 0.001.

Given the important roles of ApoE in the brain homeostasis and the association of ApoE genotypes with stroke^12^, we hypothesize that ApoE from mouse astrocytes may mediate the protective effects of conditioned medium. Thus, mouse ApoE was depleted from CM by pulldown with ApoE antibody. The CM before and after ApoE depletion was analyzed by western blot and the results showed that ApoE was completely removed from CM with ApoE antibody (Fig. 2a). In contrast, IgG had no effect on the ApoE level in CM.

Then, CM with ApoE depletion (OGD-R + CM + ApoE antibody group) or without ApoE depletion (OGD-R + CM + IgG group) was used to incubate OGD-R treated HUVEC cells. The results showed that CM with ApoE depletion failed to restore cell viability (Fig. 2b), reduce ROS level (Fig. 2c), inhibit caspase activity (Fig. 2d), or reduce apoptosis rate (Fig. 2e) in OGD-R treated HUVEC cells. In contrast, CM with IgG pulldown preserved the protective effects. It suggests that ApoE is required for the protective effects of astrocyte-derived CM.

### Astrocyte-derived CM activates PI3K/eNOS signaling in endothelial cell

**Fig. 3:**
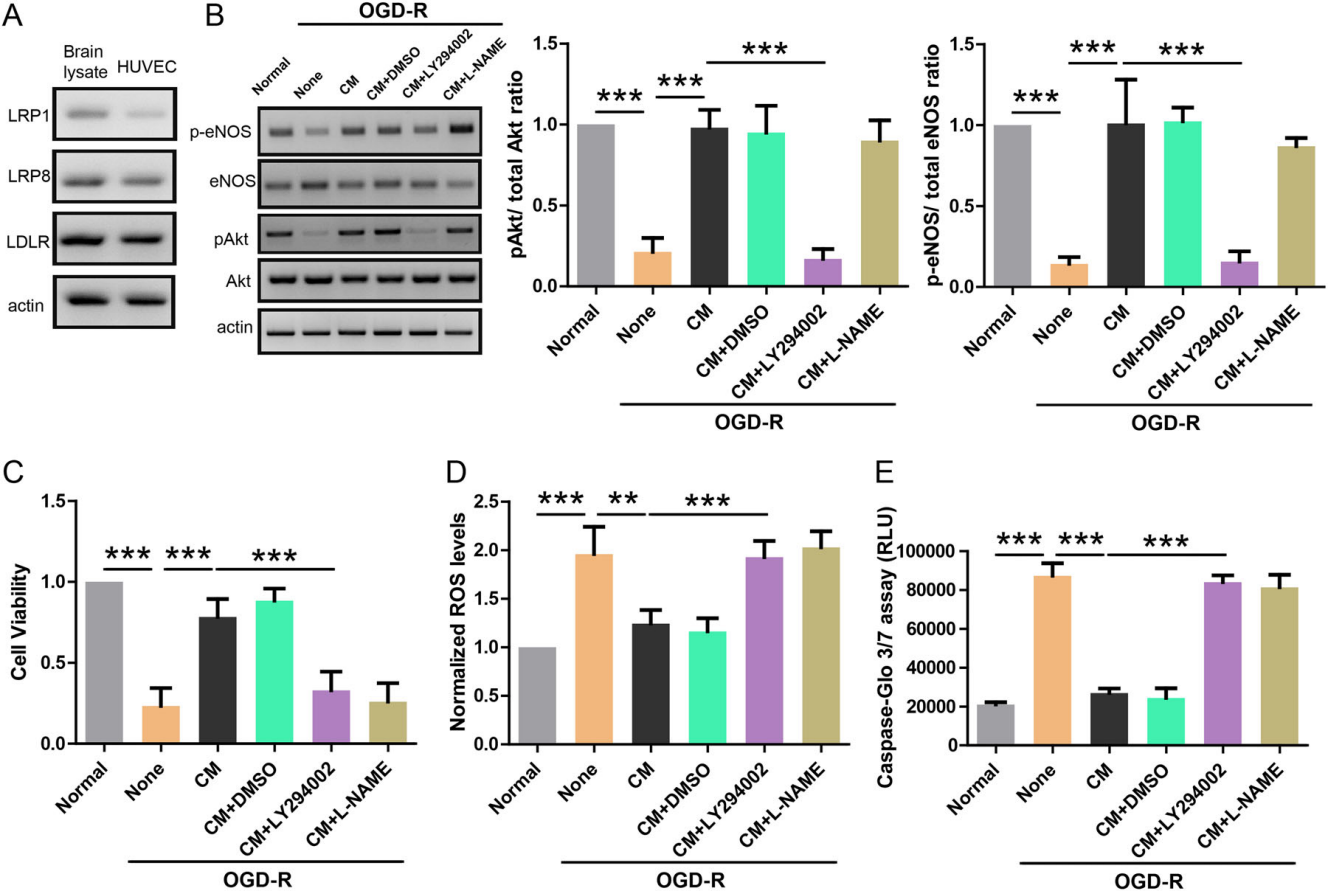
Astrocyte-derived CM activates PI3K/eNOS signaling in endothelial cell. **a** Western blot showing the protein levels of ApoE receptors (LRP1, LRP8, and LDLR) in mouse brain lysate or HUVEC cell. **b** Western blot showing the protein levels of p-eNOS, eNOS, pAkt, and Akt in endothelial cell treated with OGD-R and indicated CM in the presence of LY294002 (1 μM) or l-NAME (1 μM; *n* = 4). **c** CCK-8 assay showing the cell viability of endothelial cells treated with OGD-R and indicated CM in the presence of LY294002 (1 μM) or l-NAME (1 μM; *n* = 4). **d** ROS production in endothelial cells treated with OGD-R and indicated CM in the presence of LY294002 (1 μM) or l-NAME (1 μM; *n* = 4). **e** Caspase-Glo 3/7 assay showing caspase activity in endothelial cells treated with OGD-R and indicated CM in the presence of LY294002 (1 μM) or l-NAME (1 μM; *n* = 4). **P < 0.01, ***P < 0.001.

Then, we tried to explore the mechanism underlying the protective effects of ApoE containing CM on OGD-R treated HUVEC cell. Studies in endothelial cells have shown that ApoE could activate PI3K signaling to enhance eNOS which is essential for endothelial homeostasis^13,14^. Thus, we first determined whether HUVEC cell expressed ApoE receptors using western blot (Fig. 3a). The results showed that HUVEC cell has high expression levels of major ApoE receptors including LRP1, LRP8, and LDLR compared to mouse brain lysate which was used as positive control. Then, we incubated OGD-R treated HUVEC cell with ApoE containing medium in the presence of LY294002 (inhibitor for PI3K) or l-NAME (inhibitor for eNOS) and then measured OGD-R induced injuries in HUVEC cell. Western blot showed that OGD-R treatment reduced pAkt/ total Akt ratio as well as p-eNOS/total eNOS ratio, and ApoE containing CM fully restored these deficits (Fig. 3b). In addition, both LY294002 (1 μM) and l-NAME (1 μM) were able to block the protective effects of ApoE containing CM in CCK-8 assay (Fig. 3c), ROS assay (Fig. 3d), and Caspase-Glo 3/7 assay (Fig. 3e) on OGD-R treated HUVEC cell. These results support that PI3K/eNOS pathway mediates the protective effects of ApoE containing CM on OGD-R treated HUVEC cell.

### OGD-R induces down-regulation of ApoE in astrocyte–endothelial cell co-cultures

**Fig. 4:**
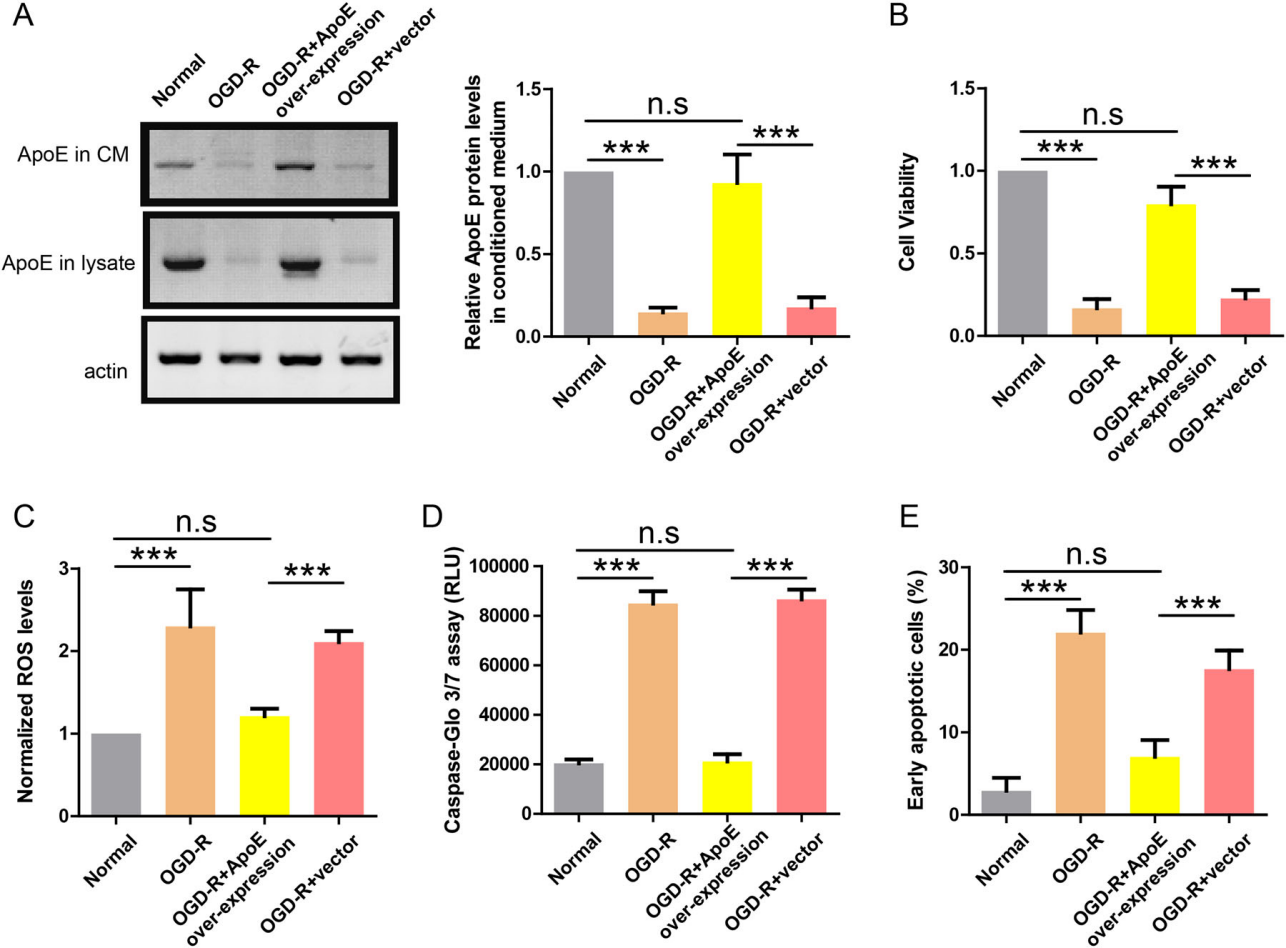
OGD-R induces down-regulation of ApoE in astrocyte-endothelial cell co-cultures. **a** Western blot showing ApoE protein levels in CM and lysate of astrocytes treated with OGD-R and indicated lenti-virus. **b** CCK-8 assay showing the cell viability of endothelial cells treated with OGD-R and indicated lenti-virus (*n* = 4). **c** ROS production in endothelial cells treated with OGD-R and indicated lenti-virus (*n* = 4). **d** Caspase-Glo 3/7 assay showing caspase activity in endothelial cells treated with OGD-R and indicated lenti-virus (*n* = 4). **e** Apoptosis rate revealed by Annexin V/PI assay in endothelial cells treated with OGD-R and indicated lenti-virus (*n* = 4). ***P < 0.001.

To fully recapitulate the in-vivo interaction between astrocytes and endothelial cells, primary astrocytes from wild-type mouse were co-cultured with HUVEC cell line in Transwell plate and the co-cultures were treated with OGD-R. The lysates and supernatants of astrocytes were analyzed by western blot and the results showed that ApoE levels in lysates and supernatants of OGD-R treated astrocytes were greatly reduced compared to normal co-cultured astrocytes (Fig. 4a). It suggests that OGD-R inhibited ApoE expression in astrocytes. Consistently, OGD-R was still able to induce injuries in HUVEC cells in co-cultures as shown by reduced cell viability (Fig. 4b), higher ROS level (Fig. 4c), enhanced caspase activity (Fig. 4d), and more apoptotic cells (Fig. 4e). To demonstrate that ApoE from astrocytes was sufficient to protect endothelial cells, astrocytes were infected with lenti-virus expressing ApoE (lenti-ApoE) before OGD-R and western blot showed that ApoE levels in the lysates and supernatants of lenti-ApoE infected astrocytes remained comparable to control astrocytes (Fig. 4a). Indeed, ApoE over-expression in astrocytes fully protected HUVEC cells from various injuries in OGD-R treated co-cultures (Fig. 4b–e). Taken together, these results again suggest that ApoE in astrocyte-derived CM mediates its protective effects on endothelial cells.

### Melatonin restores ApoE expression and protects endothelial cell in co-cultures

**Fig. 5:**
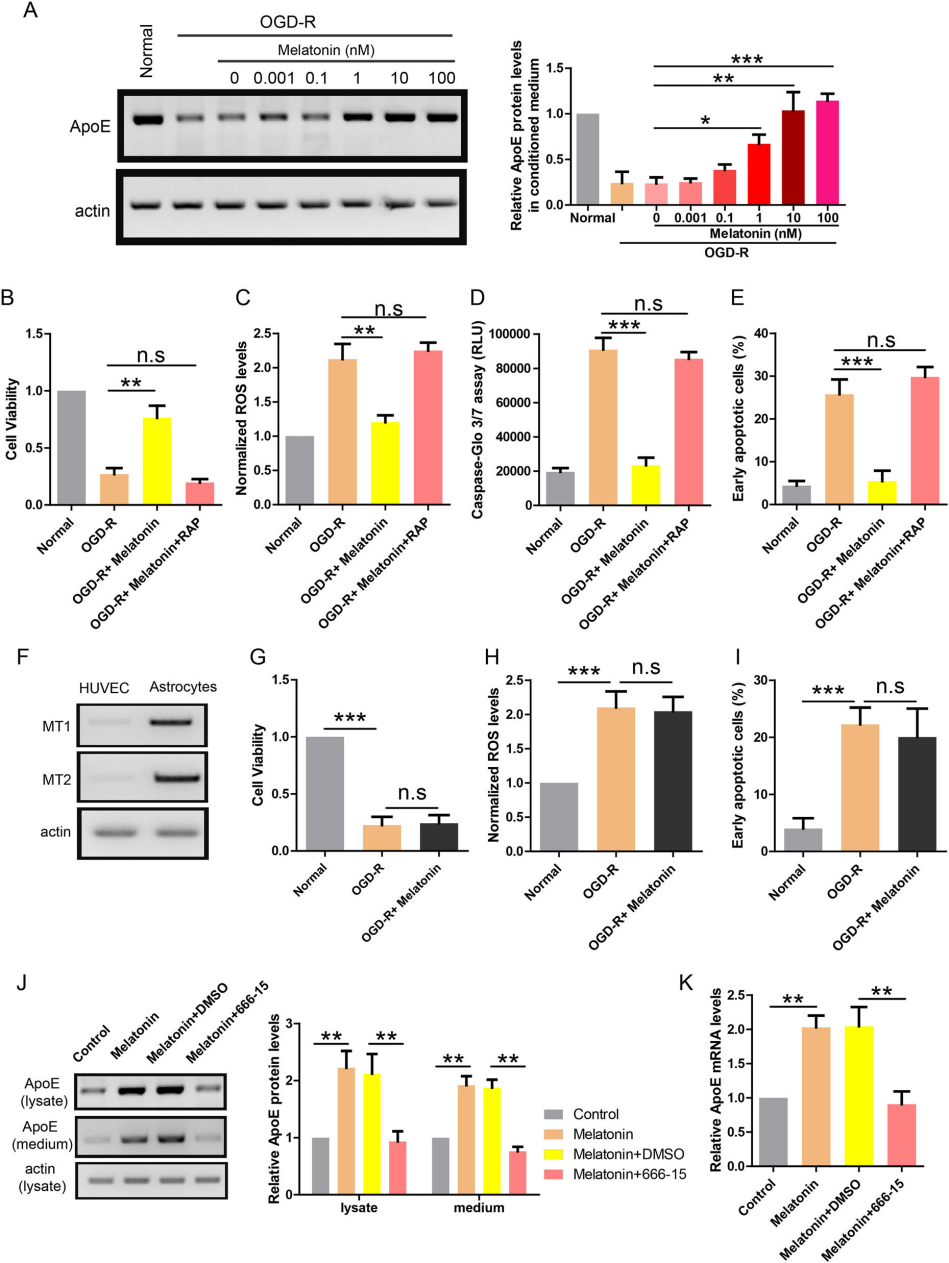
Melatonin restores ApoE expression and protects endothelial cell in co-cultures. **a** Western blot showing ApoE protein level in the lysate of astrocytes treated with OGD-R and melatonin at indicated concentrations (0, 0.001, 0.1, 1, 10, and 100 nM). **b** CCK-8 assay showing the cell viability of co-cultured endothelial cells treated with OGD-R, melatonin (1 nM) or RAP (200 nM; *n* = 4). **c** ROS production in co-cultured endothelial cells treated with OGD-R, melatonin (1 nM), or RAP (200 nM; *n* = 4). **d** Caspase-Glo 3/7 assay showing caspase activity in co-cultured endothelial cells treated with OGD-R, melatonin (1 nM), or RAP (200 nM; *n* = 4). **e** Apoptosis rate revealed by Annexin V/PI assay in co-cultured endothelial cells treated with OGD-R, melatonin (1 nM), or RAP (200 nM; n = 4). **f** Western blot showing the protein levels of melatonin receptors (MT1 and MT2) in HUVEC cell or astrocytes. **g** CCK-8 assay showing the cell viability of endothelial cells treated with OGD-R or melatonin (1 nM; *n* = 4). **h** ROS production in endothelial cells treated with OGD-R or melatonin (1 nM; *n* = 4). **i** Apoptosis rate revealed by Annexin V/PI assay in endothelial cells treated with OGD-R or melatonin (1 nM; *n* = 4). **j** Western blot showing ApoE protein levels in the lysate and medium of astrocytes treated with melatonin (1 nM) or 666-15 (0.1 μM). **k** Real-time PCR showing ApoE mRNA levels in astrocytes treated with melatonin (1 nM) or 666-15 (0.1 μM). **P < 0.01, ***P < 0.001.

Given the important roles of melatonin in the brain function and the general protective roles of melatonin in various pathological conditions^15^, we investigated the potential effects of melatonin in OGD-R treated co-cultures of astrocyte–endothelial cells. The lysates of astrocytes were analyzed by western blot and the results showed that melatonin restored ApoE expression in OGD-R treated astrocytes (Fig. 5a) in a dose-dependent manner. Consistently, melatonin (1 nM) also blocked OGD-R induced injuries in co-cultured HUVEC cells as shown by improved cell viability (Fig. 5b), lower ROS level (Fig. 5c), reduced caspase activity (Fig. 5d), and less apoptotic cells (Fig. 5e). In addition, the protective effects of melatonin were abolished by RAP (200 nM), the inhibitor of ApoE receptors. It indicates that the protective effects of melatonin in co-cultures are mediated by ApoE.

To investigate whether melatonin could directly act on endothelial cells, we first determined whether HUVEC cell expressed melatonin receptors (MT1 and MT2) using western blot. The results showed that melatonin receptors were almost absent in HUVEC cell compared to astrocytes (Fig. 5f). Then, we measured the potential effects of melatonin on HUVEC alone without co-cultured astrocytes. Consistently, the results showed that melatonin treatment had no effect on the compromised cell viability (Fig. 5g), higher ROS level (Fig. 5h), and more apoptotic cells (Fig. 5i) in OGD-R treated HUVEC cells. It suggests that melatonin is unlikely to directly act on endothelial cells. Instead, it further supports that melatonin acts on astrocytes to protect endothelial cells.

Next, we explored how melatonin regulates ApoE expression in astrocytes. Studies have shown that melatonin receptor activation enhances phosphorylation of CREB^16^ and deletion of melatonin receptors reduces pCREB levels^17^. Meanwhile, CREB could promote ApoE expression^18^. Thus, we hypothesize that melatonin induces ApoE expression through CREB pathway in astrocytes. To demonstrate this, astrocytes were treated with melatonin in the presence or absence of 666-15 (CREB inhibitor) and ApoE expression was measured by western blot (Fig. 5j) or real-time PCR (Fig. 5k). The results showed that melatonin alone increased ApoE protein and mRNA levels in astrocytes while co-treatment with 666-15 (0.1 uM) blocked this effect. These data support that melatonin induces ApoE expression through pCREB in astrocytes.

## Discussion

Although huge efforts have been made to find novel therapies for stroke, recombinant tissue plasminogen activator (rt-PA) remains the only FDA-approved treatment for patients with stroke^19^. However, rt-PA is only effective in a small portion of patients within a narrow time window. Thus, it is imperative to explore novel targets for the treatment of stroke. Here, we focus on the interaction between astrocytes and endothelial cells to provide a comprehensive understanding of brain ischemia. We find that mouse astrocytes secrete ApoE to protect endothelial cells against OGD-R induced injuries by activating PI3K/eNOS signaling. Although OGD-R inhibited ApoE expression in astrocytes, Melatonin could restore ApoE expression in astrocytes via pCREB pathway and protect endothelial cells from OGD-R induced injuries. Thus, targeting the interaction between astrocytes and endothelial cells might provide a new avenue to treat stoke.

ApoE is expressed in several organs with the highest expression in the liver, followed by the brain. In general, the wild-type ApoE3 plays essential and protective roles in the brain. However, the ApoE4 mutant compromises its normal function and ApoE4 has been implicated in Alzheimer’s disease, impaired cognitive function, reduced hippocampal volume, faster disease progression in multiple sclerosis, poor prognosis of traumatic brain injury, and reduced neurite outgrowth^20^. Interestingly, ApoE4 genotype is also associated with stroke and its underlying mechanism remains unclear. There are studies reporting that APOE4 genotype is associated with poor recovery of stroke^21^, younger onset age of stroke^22^ and cognitive impairment after stroke^23^. There are also meta-analysis studies showing that APOE4 genotype is significantly associated with ischemic stroke^24,25^. All these clinical studies support that ApoE is involved in the pathogenesis of ischemic stroke and our study reveals how ApoE mediates the cross-talk between astrocytes and endothelial cells under the condition of ischemic stroke. It is important to note that we used mouse astrocytes to investigate the function of mouse ApoE in our study. We show that asctrocyte-derived ApoE is protective for OGD-R treated endothelial cells and OGD-R resulted in the loss of expression and function of ApoE in astrocytes. As wild-type mouse ApoE is equivalent to human ApoE3, it is reasonably to speculate that human ApoE3 may share similar effects as mouse ApoE and human ApoE4 may increase stroke risk possibly by compromising the ApoE-mediated astrocyte–endothelial cell interaction. It would be of great interest to study the isoform-specific effects of human ApoE in future studies.

Melatonin is primarily produced by the pineal gland in mammals. Its secretion, mainly at night, is regulated in a circadic manner by the suprachiasmatic nucleus in response to environmental light–dark cycles^26^. Melatonin has been known to exert direct and indirect antioxidant actions both at physiologic, endogenous concentrations, as well as at concentrations exceeding several orders of magnitude the physiologic levels^27^. Melatonin has numerous protective roles as it could inhibit tumor cell aggression^28,29^ and protect cardiomyocyte^30^. The protective effects of melatonin on neuron have been reported in stroke models. However, it remains unclear whether and how melatonin cloud act on astrocyte and endothelial cells. Here, we show that melatonin could protect OGD-R treated endothelial cells through acting on ApoE from astrocytes. There are several clinical studies reporting that melatonin can help to recover from stroke^31–33^. These clinical studies are fully consistent with our current findings and our study reveals a potential molecular mechanism underlying the protective effects of melatonin in human patients. Taken together, our study further supports that melatonin may be a effective treatment to stroke as it could protect multiple cell types involved in ischemia brain damages.

In summary, we report that melatonin-induced ApoE expression in astrocytes protects endothelial cells from OGD-R induced injuries. Thus, targeting the interaction between astrocytes and endothelial cells might provide a new avenue to treat stoke.
