# Correction: Melatonin-induced ApoE expression in mouse astrocytes protects endothelial cells from OGD-R induced injuries

**DOI:** 10.1038/s41398-020-00925-z

**Published:** 2020-07-17

**Authors:** Jun Xiang, Wen Zhu, Feng Yang, Zhong-Hai Yu, Min Cai, Xiang-Ting Li, Jing-Si Zhang, Wen Zhang, Ding-Fang Cai

**Affiliations:** 1 Department of Integrative Medicine, Zhongshan Hospital, Fudan University, 200032 Shanghai, China; 2 Laboratory of Neurology, Institute of Integrative Medicine, Fudan University, 200032 Shanghai, China; 3 Department of Traditional Chinese Medicine, the Sixth People’s Hospital Affiliated to Shanghai Jiao Tong University, 200233 Shanghai, China; 4 Department of Neurology, Shuguang Hospital Affiliated to Shanghai University of Traditional Chinese Medicine, 201203 Shanghai, China

Correction to: *Translational Psychiatry*

10.1038/s41398-020-00864-9 published online 8 June 2020

This Article was updated shortly after publication in order to amend co-author Jing-Si Zhang’s affiliations (Dr Zhang was erroneously associated with affiliations 1 to 4 rather than just affiliation 1 as is correct) and various typographical errors in superscripts and subscripts.

